# Evaluation of EGFR inhibitor‐mediated acneiform skin toxicity within the double‐blind randomized EVITA trial: A thorough gender‐specific analysis using the WoMo score

**DOI:** 10.1002/cam4.2132

**Published:** 2019-06-14

**Authors:** Maria R. Gaiser, Sylvie Lorenzen, Kirsten Merx, Jörg Trojan, Janja Ocvirk, Thomas J. Ettrich, Salah‐Eddin Al‐Batran, Holger Schulz, Nils Homann, Hans‐Peter Feustel, Michael Schatz, Melanie Kripp, Nadine Schulte, Steffen Heeger, Soetkin Vlassak, Winfried Koch, Ralf‐Dieter Hofheinz

**Affiliations:** ^1^ Department of Dermatology, Venereology and Allergology University Medical Center Mannheim, Ruprecht‐Karl University of Heidelberg Mannheim Germany; ^2^ Skin Cancer Unit German Cancer Research Center (DKFZ) Heidelberg Germany; ^3^ Medical Clinic III University of Munich Munich Germany; ^4^ Interdisciplinary Tumor Center Mannheim University Medical Center Mannheim, Ruprecht‐Karl University of Heidelberg Mannheim Germany; ^5^ Medical Clinic I University Hospital Frankfurt Frankfurt Germany; ^6^ Institute of Oncology Ljubljana Slovenia; ^7^ Medical Clinic I University Hospital Ulm Ulm Germany; ^8^ Institute of Clinical Cancer Research (IKF) at Nordwest Hospital UCT‐University Cancer Center Frankfurt Germany; ^9^ Oncological Practice Frechen Germany; ^10^ Medical Clinic II Wolfsburg Wolfsburg Germany; ^11^ Hämatologisch‐Onkologische Praxis Speyer Germany; ^12^ Medizinische Klinik II, ViDia Christliche Kliniken Karlsruhe Karlsruhe Germany; ^13^ Mannheim Germany; ^14^ Neervelp Belgium; ^15^ BDS Koch Schwetzingen Germany

**Keywords:** acneiform skin toxicity, EGFR inhibitor, women, WoMo score

## Abstract

Acne‐like skin reactions frequently occur in patients undergoing treatment with drugs inhibiting the epidermal growth factor receptor. Recently, the effects of vitamin K1 containing cream (Reconval K1) as prophylactic skin treatment in addition to doxycycline were explored in a double‐blind randomized phase II trial (EVITA) in patients with metastatic colorectal cancer receiving cetuximab. EVITA demonstrated a trend towards less severe skin rash in Reconval K1‐treated patients using the tripartite WoMo skin reaction grading score as a thorough tool for quantification of drug related skin reactions. This gender‐specific analysis of the EVITA trial evaluated the application of the WoMo score for assessment of epidermal growth factor receptor (EGFR)‐related skin toxicities according to treatment arm and gender. To show the robustness of results parametric and non‐parametric statistical analyses were conducted. All three parts of the WoMo score independently demonstrated the superiority of the treatment arm (Reconval K1) regarding a significant reduction in acneiform skin reactions in women. Men did not benefit from Reconval K1 cream at any time point in none of the WoMo score analyses. The treatment effect in women was confirmed by the use of skin rash categories based on the final WoMo overall score and mixed effect longitudinal multiple linear regression analysis. The WoMo score represents a sensitive tool for studies exploiting treatments against EGFR mediated acne‐like skin rash. Part C of the WoMo score seems to be sufficient for quantification of drug related skin toxicities in further studies. Standard WoMo skin reaction score values for future studies are provided.

## 1 INTRODUCTION

Treatment with drugs inhibiting the epidermal growth factor receptor (EGFR) often causes skin toxicities within the first weeks of treatment.^1^ These skin reactions can lead to physical and psychosocial discomfort affecting treatment adherence and clinical outcome.^2^ Special skin care and tetracyclines are used as prophylactic treatment,^3^ but still a high percentage (20%‐30%) of patients treated with drugs targeting EGFR suffer from NCI‐CTC ≥ grade 2 skin toxicities.^2^, ^4^

Recently, Reconval K1, a vitamin K1 containing cream as an adjunct to doxycycline was therefore compared to the vehicle (Reconval) in an investigator‐initiated double‐blind, randomized phase II trial (EVITA, clinical trial number NCT01345526) as prophylactic treatment for skin rash in patients receiving first line cetuximab‐based treatment for metastatic (K)RAS wild type colorectal cancer.^5^ Stratification in this trial was done by gender and participating center. The primary endpoint of the EVITA trial, improvement of acneiform skin rash ≥grade 2 according to NCI CTC criteria, was not met, but a significantly reduced risk of rash in women versus men was noticed (Odds ratio 0.15; 95% CI, 0.06‐0.36). Moreover, by exploring skin rash with the more thorough tripartite skin grading score for the evaluation of acneiform skin eruptions induced by EGFR inhibition that has been developed by Wollenberg and Moosmann (WoMo score)^6^ (the main secondary endpoint in the EVITA trial), less severe skin toxicity (measured with part C of this score) was found in patients treated with Reconval K1 cream. This effect increased over time during the 8 weeks of skin treatment and was still present in patients followed‐up until week 12.

In this posthoc analysis of the EVITA trial we sought to explore the effects of Reconval K1 cream according to gender by using the WoMo score. Preliminary data of one segregated part of the statistical analysis (the so‐called WoMo final score) were recently reported indicating that women derived a benefit from the use of Reconval K1 cream in terms of the overall WoMo score while men did not.^7^ Herein, we report the full comprehensive dataset, including the in‐depth analysis of all parts of the WoMo score along with confirmatory statistical modeling and data on treatment adherence according to gender. Moreover, we provide WoMo values over time as basis for planning future trials and assess the potential of any of the 3 parts of the WoMo score for the purpose of a simplification of the WoMo score.

## 2 PATIENTS AND METHODS

An exploratory analysis of data from the EVITA study was conducted. EVITA was a randomized, double‐blind, vehicle‐controlled phase II investigator‐initiated clinical trial (clinical trials number NCT01345526) in patients with metastatic (K)RAS wild type colorectal cancer treated with FOLFIRI plus cetuximab as first line treatment.^5^ All patients received doxycycline as a prophylaxis at a dose of 100mg bid for a total of 8 weeks. Local skin treatment comprised vitamin K1 containing cream (Reconval K1) 0.1% or the respective vehicle (Reconval) bid for 8 weeks, as well. Skin toxicities were evaluated on a weekly basis over the 8 weeks of treatment followed by an observation period of 4 weeks using NCI CTC criteria and WoMo grading (Supplementary Table S1).^6^ WoMo includes 3 parts. Part A reflects on the affected body surface, part B describes the facial involvement, and part C involves the severity of rash. Moreover, it allows for determination of an overall score and a categorization according to the points achieved with the skin rash grading.^6^ The primary endpoint of the study was the incidence of grade ≥2 skin rash (NCI CTC version 4.02) during 8 weeks of skin treatment. A key secondary end point comprised skin rash grading according to WoMo score. WoMo part A corresponds to the NCI CTC criteria for grading skin rash which reflects the percentage of the affected body surface. WoMo part B describes the percentage of the face affected by skin rash, while WoMo part C describes the severity of the rash (Supplementary Table S1).

The current exploratory analysis focused on the evolution of WoMo values (parts A, B, C) over time according to gender with a special focus on WoMo part C (ie on the severity of skin rash). To exclude gender‐specific compliance differences, we analyzed the adherence to study treatment by gender (ie Reconval K1 cream or vehicle and doxycyline) as well as the use of anti‐cancer drugs (especially cetuximab).

Statistical methods for the current analysis were not predefined in the study protocol. Several parametric and non‐parametric approaches were used in the sense of an exploratory data analysis. To take repeated measures and missing data (especially in the second part of the observation period, ie after week 5) into account, mixed effect longitudinal multiple linear regression was applied to model square root (Sqrt) (WoMo C) by gender as a function of week, drug, and week*drug interaction including patient as a random effect. Week as main effect was included as a continuous variable where a 4‐knot spline was found most appropriate to ensure sufficient flexibility for fitting the time course of WoMo C. Because of the skewness of the distribution of WoMo C and its truncation at 0, the Sqrt transformation was applied for statistical modeling. Mean time course of WoMo variables (not transformed) is presented by gender and treatment. In addition to these parametric analyses, simple non‐parametric analyses were conducted using the Wilcoxon Rank‐Sum test to compare the WoMo levels over the course of the observation period at each visit. The pattern of resulting *P*‐values is discussed in the sense of exploratory data analysis. Moreover, an analysis of the application of the WoMo skin toxicity grading categories (ie no rash, mild, moderate, or severe rash; for details see Supplementary Table S1) over time and according to gender was done.

Statistical analyses were performed using SAS JMP V13.2.1 (SAS Institute, Cary, NC, USA).

## 3 RESULTS

A total of n = 126 patients were included in the primary analysis of the EVITA trial. Four of these patients could not be included in the current analysis due to missing post‐baseline WoMo values leaving a total of n = 122 patients for the analysis (n = 85 men and n = 37 women). The main characteristics are depicted in Table 1.

**Table 1:** Patient characteristics

Patients	Vitamin K	Vehicle
All (N = 122)	60	62
Median age (range)	63 (44‐83)	63 (24‐84)
WHO performance status		
0 (N = 72)	34	38
1 (N = 50)	26	24
Female (N = 37)	18	19
Median age (range)	61 (45‐80)	67 (43‐77)
WHO performance status		
0	12	12
1	6	7
Male (N = 85)	42	43
Median age (range)	64 (44‐83)	62 (24‐84)
WHO performance status		
0	22	26
1	20	17

### 3.1 Compliance with study treatment and anti‐cancer treatment

Gender‐specific analyses including treatment duration and cumulative doses of anti‐cancer drugs administered as well as adherence to the prophylactic skin treatment (ie doxycycline and Reconval K1/vehicle cream) during the 8‐week skin treatment period are provided in Supplementary Table S2a and Sb for female and male patients, respectively. No relevant differences in treatment duration and administered doses of anti‐cancer drugs were observed between treatment arms in men and women.

Comparably, for both, men and women, the median and cumulative numbers of daily administrations of doxycycline and Reconval K1/vehicle creams were virtually identical.

### 3.2 Evolution of WoMo C values over time according to gender

**Figure 1:**
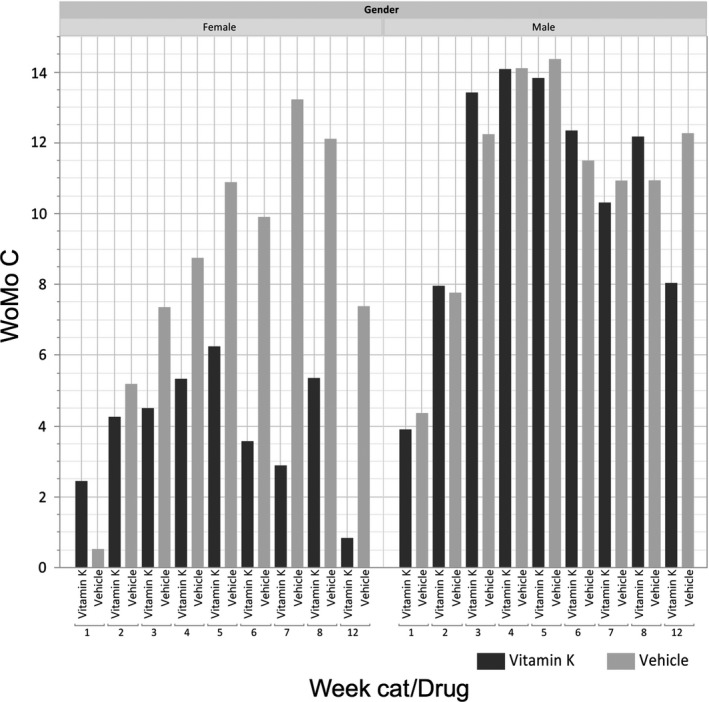
Observed mean values for WoMo C score according to gender (women: left part; men: right part), allocated treatment arm (Vitamin K: dark grey bars, vehicle: light grey bars), and treatment week

Figure 1 depicts the observed mean values of WoMo C according to gender (left part of the plot women), treatment arm (dark gray bars: Vitamin K (Reconval K1) cream; light gray: vehicle), and week of treatment. The figure illustrates the typical time course of skin rash including deterioration (corresponding to higher values) during the first week and an improvement of rash starting about 6‐8 weeks after the start of treatment. WoMo C values for women in the experimental arm are clearly lower compared to the vehicle. This effect is observed repeatedly on a weekly basis; it starts at week 2 and increases over time. Contrarily, for men, no effect of Reconval K1 cream was seen at any time point (right side of the plot). The Wilcoxon tests by visit show superiority of Reconval K1 cream for female patients over vehicle from week 5 to week 12 (*P* < 0.05) while no statistically significant difference between arms was found for the male patients on any time point (Supplementary Table S3).

**Figure 2:**
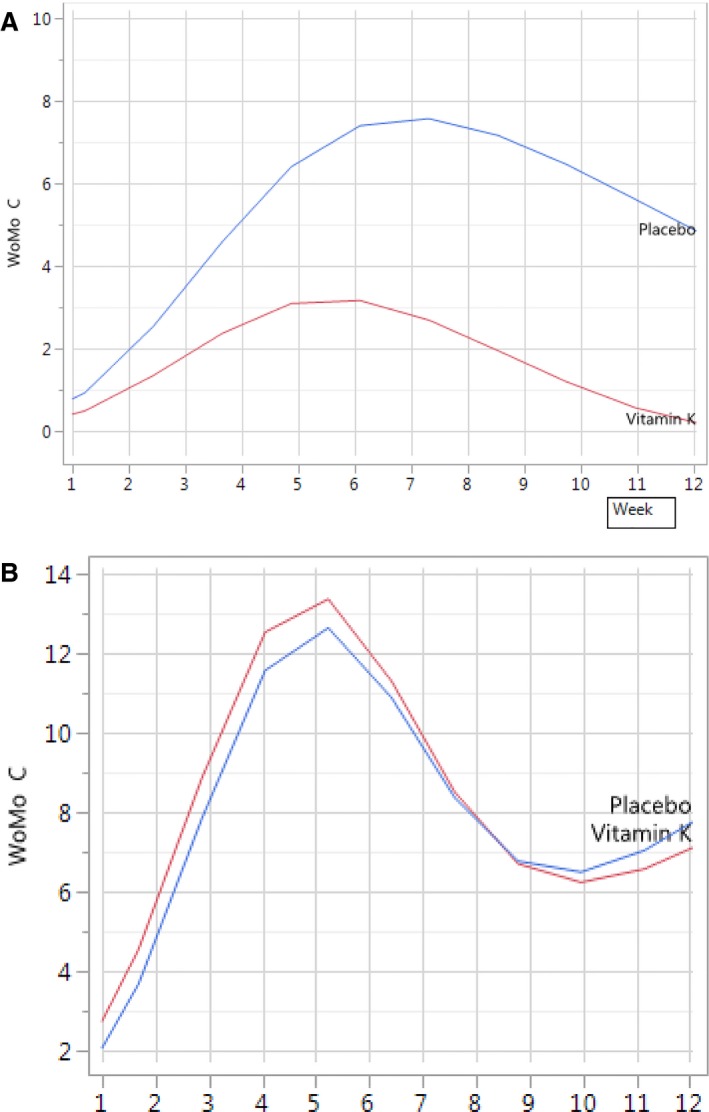
(A) Model estimated time course of WoMo C by treatment (*women*). (B) Model estimated time course of WoMo C by treatment (*men*)

Figure 2A,B show the model estimated time course of WoMo C by treatment over the observation period of 12 weeks for females and males, respectively. In the female subgroup Reconval K1 demonstrates a clear treatment benefit. The fixed effect tests depict a statistically significant interaction term Week*Drug (*P* = 0.005). The treatment effect of Reconval K1 cream is visible at an early time point but clearly emerges over time (Figure 2A). A maximum is approached at week 6 and reached at week 7. These findings are consistent with the statistical test result for the drug main effect for women which showed a *P* value of *P* = 0.1126 indicating a statistical trend mainly supported by the later phase of the observation period.

For the subgroup of male patients no treatment effect is seen during the 12‐week observation period (Figure 2B; *P* = 0.3392 for week*drug interaction term and *P* = 0.6190 for the drug main effect).

### 3.3 Evolution of WoMo B and C scores over time according to gender

Supplementary Figures S1‐S2 show the results of mean values for WoMo A and B according to gender, allocated treatment arm and week of treatment. All of these analyses paint the same picture: The plots for WoMo A and B compare very well with what is observed in the WoMo C analysis (Figure 1), namely a clear treatment effect for female patients who are statistically significant from week 5 onwards. None of these 3 analyses indicates an effect of Reconval K1 cream in male patients at any time point during the 12‐week observation period.

**Figure 3:**
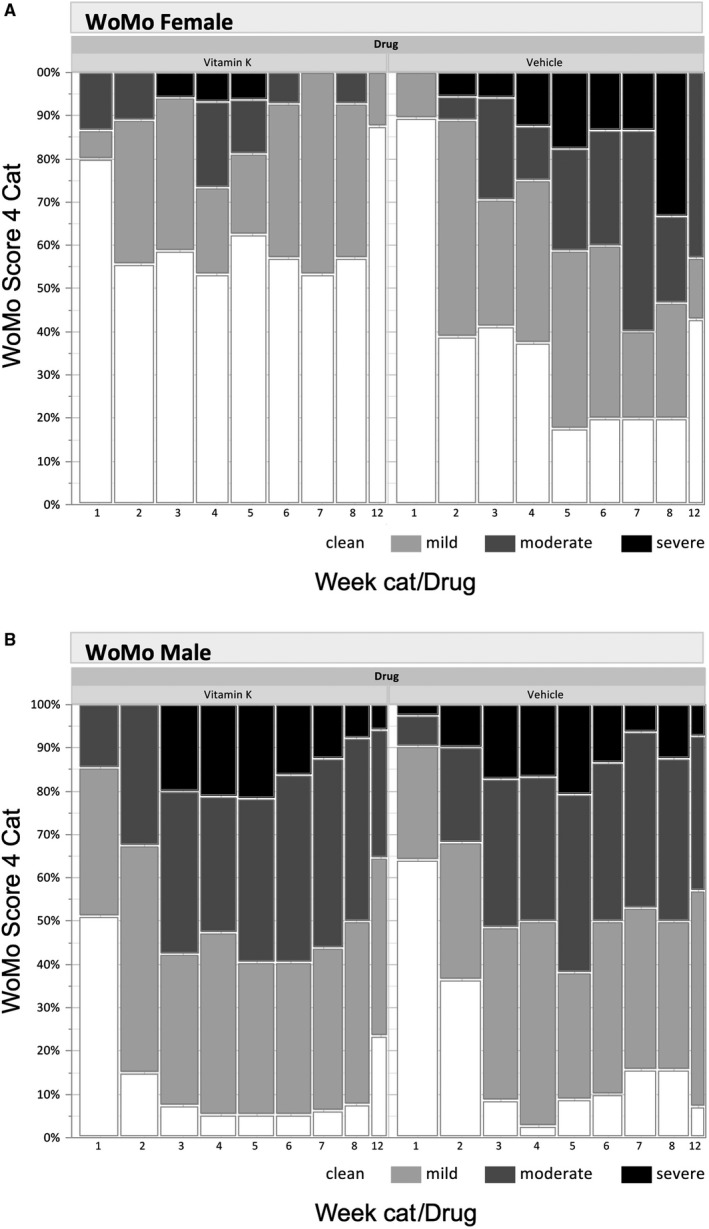
Evolution of categories of WoMo score according to treatment arm in *female* patients (A) and *male* patients (B). *Note*: Each bar indicates the percentage of patients with values for the respective week. White bars represent patients without rash (zero points), light grey bars indicate mild rash (1‐20 points), dark gray bars indicate moderate rash (21‐40 points), and black bars indicate severe rash (>40 points). The width of bars is proportional to the number of patients evaluated for the corresponding week and treatment

The benefit of using Reconval K1 is also demonstrated in terms of the evolution of categories of WoMo score according to treatment arm and gender. The original WoMo score categorizes skin rash as mild, moderate, and severe according to the number of points (mild rash 0‐20, moderate 21‐40, and severe rash > 40 points). The results according to categories and gender are depicted in Figure 3A,B, respectively. In the current data analysis, patients without rash in a week are shown as a separate category colored in white (“no rash”, ie 0 points). For female patients there are statistically significant treatment effects of Reconval K1 from week 5 to week 12. For male patients no differences and not even trends were found. The corresponding effect size estimates for female patients between weeks 5 and 12 are in the range of 1.65‐2.46 (data not shown).

## 4 DISCUSSION

The current exploratory gender‐specific analysis of the EVITA trial was done in order to evaluate the application of the tripartite WoMo score on the development of skin rash in patients receiving cetuximab‐based therapy as first line treatment for metastatic colorectal cancer. In the original EVITA trial the primary endpoint – incidence of grade ≥2 skin rash according to NCI CTC criteria – was not reached.^5^ Instead of the NCI CTC criteria, mostly reflecting on the percentage of the affected body surface, in the EVITA trial the more thorough WoMo skin rash grading score had been used as main secondary endpoint. The use of the WoMo score in the EVITA trial demonstrated that patients treated with Reconval K1 cream had less severe rash starting in week 2, reaching a statistically significant effect at week 5, further increasing over time. Moreover, female patients were found to have a lower risk for developing rash compared to men and derived a clinically significant benefit from the treatment with Reconval K1 in terms of the WoMo final score.^5^, ^7^

The major results of the in‐depth gender‐specific evaluation of the WoMo score presented herein are the consistently decreased mean values for all WoMo parts (A, B, and C) in women treated with Reconval K1 compared to the vehicle. The consistent impact of Reconval K1 cream on ameliorating the values of all 3 WoMo parts is striking, particularly because for men an effect of Reconval K1 cream was not seen on any of the 3 WoMo parts at any time point. The observed effect was verified by modeling these data for WoMo part C using mixed effect longitudinal multiple linear regression analysis. Thus, an overall treatment effect as reported for the WoMo final score^7^ is reflected by all parts of the WoMo scoring. Thus, it might be discussed if the use of part C only is adequate for future studies.

The breakdown of the WoMo categories demonstrated a comparable high number of women developing no rash at all when treated with both, doxycycline and Reconval K1 cream, while in contrast, only a minority of men presented without signs of skin rash from week 2 on.

The restriction of positive effects of Reconval K1 cream on female skin only could have several reasons. An important factor could be a better compliance of females in terms of a correct use of the topical applicable Reconval K1 cream. For instance, it has been reported that men are less likely to use sunscreen^8^ and that men generally have a worse treatment adherence than females.^9^, ^10^ The same might apply for the use of other creams that have to be applied on a regular basis. We have performed a gender‐specific analysis of the dose intensity of the study drugs administered as well as the dose‐intensity of the chemotherapy. We found no difference for the use of Reconval K1 or vehicle cream application for both sexes. Likewise, the treatment duration and cumulative doses of cetuximab administered were almost identical between allocated treatment arms for man and women. Thus, gender‐specific different adherences to study treatment or imbalances between arms in terms of the use of cetuximab are unlikely.

The positive effects of vitamin K containing cream on anti‐EGFR drug‐related acneiform skin rash are based on an EGFR (re)activation on keratinocytes counteracting the EGFR inhibitory effects caused by the systemic anti‐EGFR treatment.^11^ We previously demonstrated a comparable anti‐tumor effect of anti‐cancer medication in both treatment arms. The response rates and the progression‐free survival results were not different indicating that no systemic vitamin K effect could be observed impacting on patients' outcome^5^ indicating that the effects of vitamin K are limited to the skin and do not interfere with the required anti‐EGRF related effects on cancer cells.

Moreover, gender‐specific dimorphisms regarding the hormonal modulation of EGFR could cause the better effect of Reconval K1 on females. It has been demonstrated that sexual hormones like estrogen and testosteron can influence pro‐EGF and EGF receptor mRNA levels, thus modulating EGFR concentration and EGFR binding.^12^ Furthermore, men have a terminal character of facial hair and a higher density of sebaceous glands, both probably causing a higher density of EGFR expression. And last, due to the different skin morphology the transdermal delivery of active components into male skin is likely to be attenuated compared to female skin.^13^ It is therefore conceivable that dosage and/or frequency of administration of Reconval K1 cream were insufficient to observe an effect in men.

We acknowledge that a retrospective exploratory analysis has inherent shortcomings and the statistical methods for the current analysis were not pre‐specified in the study protocol. The subgroup of female patients analyzed herein may be regarded as rather small to draw firm conclusions and confirmatory studies might be considered. However, the statistical significances of the observed benefit of the study drug in all analyses of the different parts of the WoMo score justify the recommendation of an application of the WoMo score in further studies.

## 5 CONCLUSION

The WoMo score represents a sensitive tool for studies exploiting treatments against EGFR mediated acne‐like skin rash. Part C of the WoMo score seems to be sufficient for a valuable evaluation of skin toxicities.

## CONFLICT OF INTEREST STATEMENT

MRG has received honoraria from Roche, MSD, BMS, Merck KGaA, Novartis, Pierre Fabre. S‐E A‐B has an advisory role with Merck, Roche, Celgene, Lilly, Nordic Pharma, Bristol‐Myers Squibb, and MSD Sharp & Dohme; is a speaker for Roche, Celgene, Lilly, Nordic Pharma, AIO gGmbH, MCI, promedicis, and Forum für Medizinische Fortbildung; He is CEO/founder of IKF Klinische Krebsforschung GmbH and has received research grants from Sanofi, Merck, Roche, Celgene, Vifor, Medac, Hospira, Lilly, German Cancer Aid (Krebshilfe), German Research Foundation and the Federal Ministry of Education and Research. RDH has received honoraria from Merck KGaA, Amgen, Roche, MSD, BMS, medac, Lilly, Sanofi, Boehringer. He has served as an advisor for Amgen, Merck KGaA, Roche, MSD, BMS, medac, Lilly, Sanofi, Boehringer. His institution has received research funding from Amgen, Merck KGaA, Amgen, Sanofi. All remaining authors have declared no conflict of interest.
