# Supplementary figures and images for: Evaluation of EGFR inhibitor‐mediated acneiform skin toxicity within the double‐blind randomized EVITA trial: A thorough gender‐specific analysis using the WoMo score
Source: Cancer Med. 2019 Jun 14;8(9):4169–75. doi: 10.1002/cam4.2132 (PMC6675717; doi:10.1002/cam4.2132)

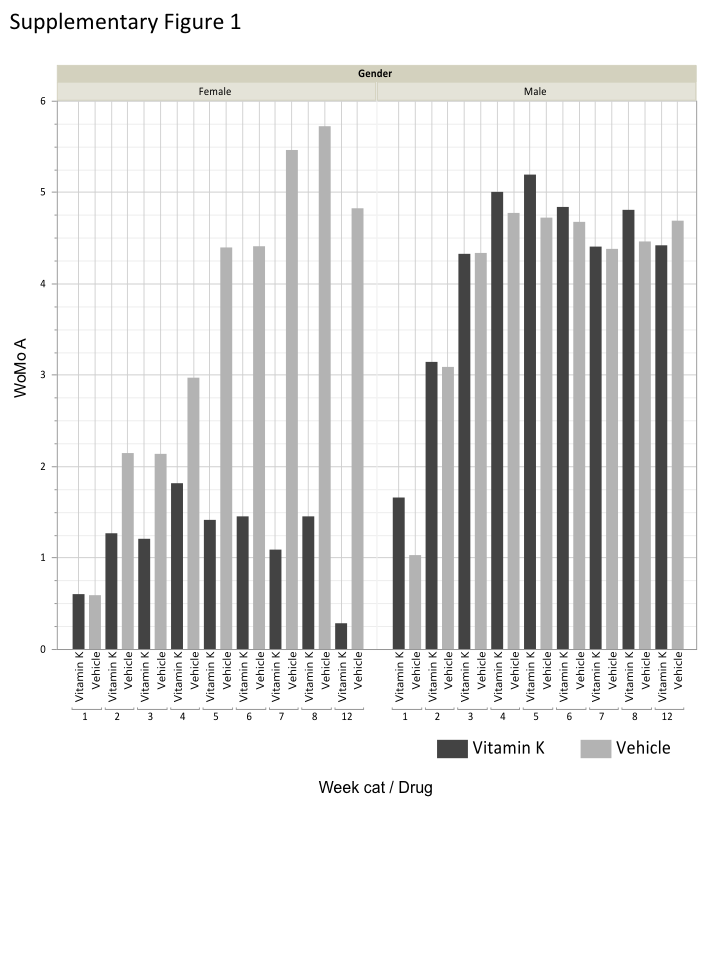

Supplement: Supplementary file 1 [file CAM4-8-4169-s001.tiff]

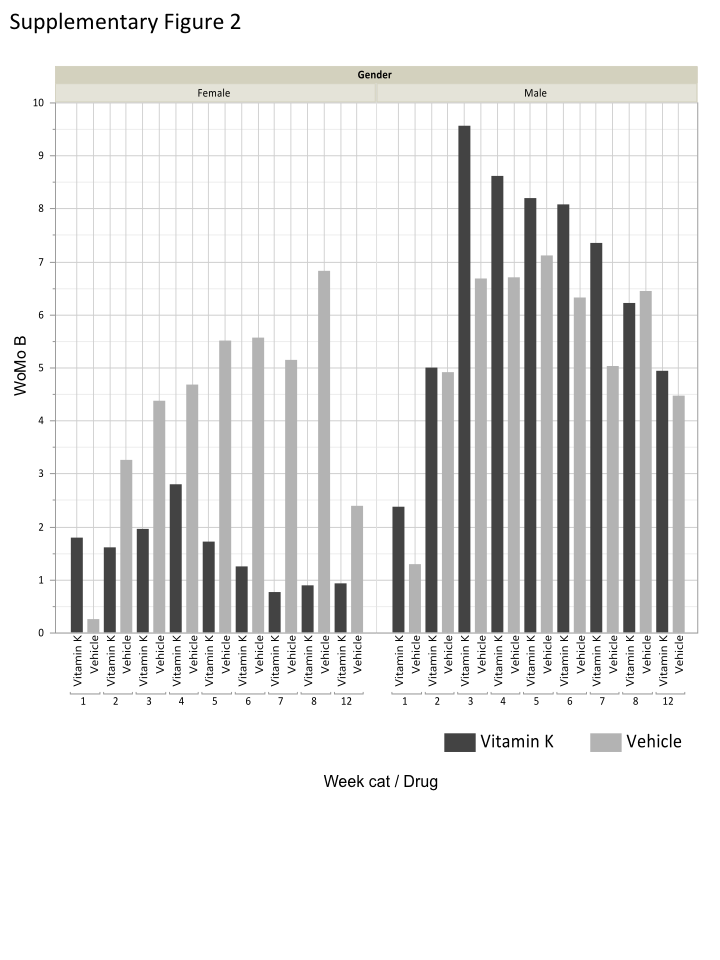

Supplement: Supplementary file 2 [file CAM4-8-4169-s002.tiff]

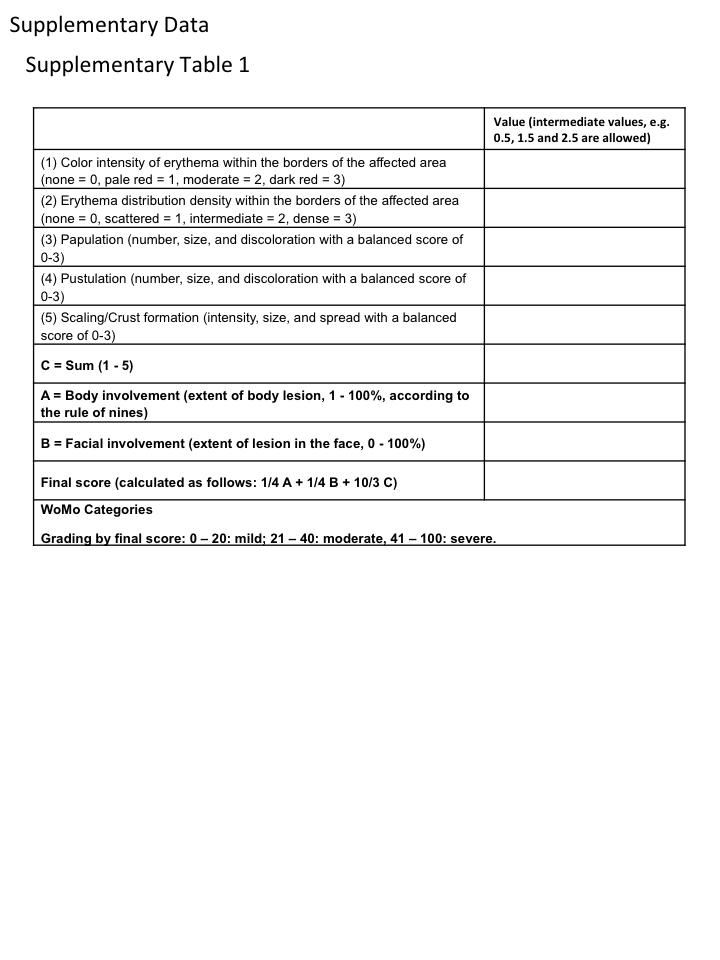

Supplement: Supplementary file 3 [file CAM4-8-4169-s003.tiff]

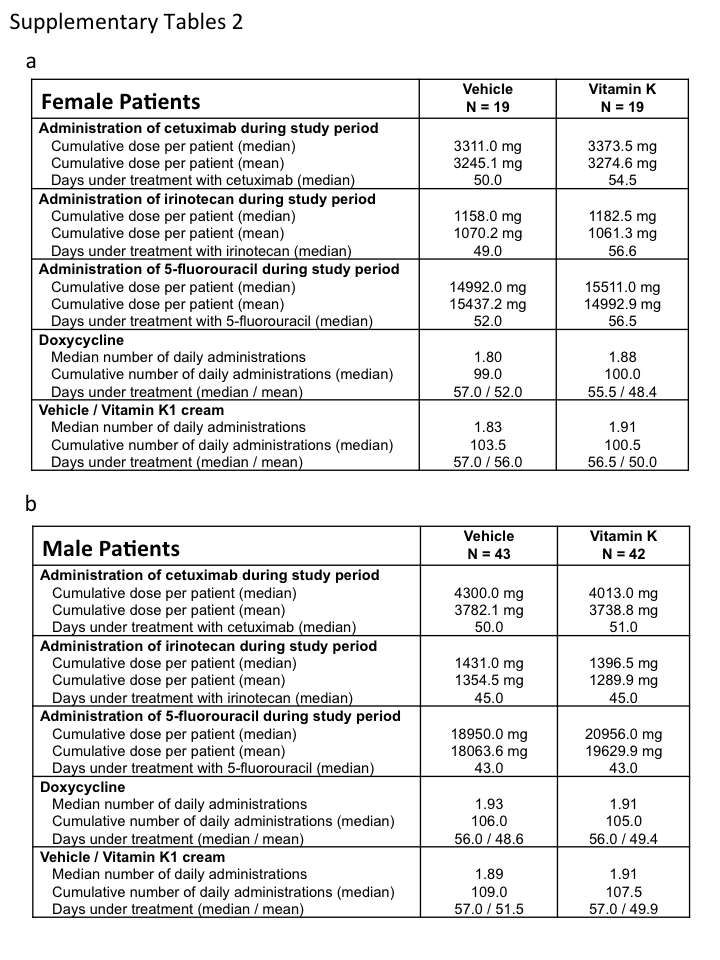

Supplement: Supplementary file 4 [file CAM4-8-4169-s004.tiff]

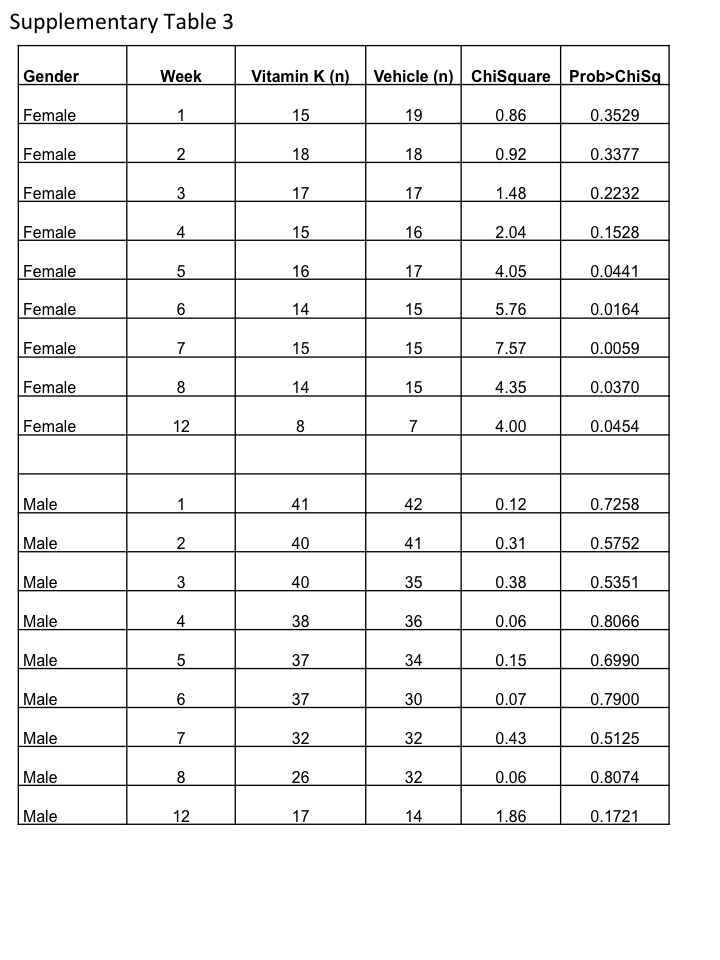

Supplement: Supplementary file 5 [file CAM4-8-4169-s005.tiff]
